# B-arrestin-2 Signaling Is Important to Preserve Cardiac Function During Aging

**DOI:** 10.3389/fphys.2021.696852

**Published:** 2021-08-27

**Authors:** Andrielle E. Capote, Ashley Batra, Chad M. Warren, Shamim A. K. Chowdhury, Beata M. Wolska, R. John Solaro, Paola C. Rosas

**Affiliations:** ^1^Department of Physiology & Biophysics, Center for Cardiovascular Research, University of Illinois at Chicago, Chicago, IL, United States; ^2^Department of Medicine, Division of Cardiology, Center for Cardiovascular Research, University of Illinois at Chicago, Chicago, IL, United States

**Keywords:** β-arrestin-2, cardiac dysfunction, angiotensin, cardiac myosin binding protein-C, myosin regulatory light chain

## Abstract

Experiments reported here tested the hypothesis that β-arrestin-2 is an important element in the preservation of cardiac function during aging. We tested this hypothesis by aging β-arrestin-2 knock-out (KO) mice, and wild-type equivalent (WT) to 12–16months. We developed the rationale for these experiments on the basis that angiotensin II (ang II) signaling at ang II receptor type 1 (AT1R), which is a G-protein coupled receptor (GPCR) promotes both G-protein signaling as well as β-arrestin-2 signaling. β-arrestin-2 participates in GPCR desensitization, internalization, but also acts as a scaffold for adaptive signal transduction that may occur independently or in parallel to G-protein signaling. We have previously reported that biased ligands acting at the AT1R promote β-arrestin-2 signaling increasing cardiac contractility and reducing maladaptations in a mouse model of dilated cardiomyopathy. Although there is evidence that ang II induces maladaptive senescence in the cardiovascular system, a role for β-arrestin-2 signaling has not been studied in aging. By echocardiography, we found that compared to controls aged KO mice exhibited enlarged left atria and left ventricular diameters as well as depressed contractility parameters with preserved ejection fraction. Aged KO also exhibited depressed relaxation parameters when compared to WT controls at the same age. Moreover, cardiac dysfunction in aged KO mice was correlated with alterations in the phosphorylation of myofilament proteins, such as cardiac myosin binding protein-C, and myosin regulatory light chain. Our evidence provides novel insights into a role for β-arrestin-2 as an important signaling mechanism that preserves cardiac function during aging.

## Introduction

The prevalence of heart failure (HF) is expected to increase due to the older population continuously growing (Virani et al., 2021). As of 2019, 6.2million Americans were afflicted with HF (Benjamin et al., 2019). Heart failure progression is associated with diastolic and systolic abnormalities which in turn are associated with maladaptive phosphorylation status of sarcomere proteins. Over activation of AT1R by ang II has been implicated in the development of hypertension during aging (Mattson and Maudsley, 2009). Ang II receptor type 1-deficient mice had a longer life span, developed less cardiac and vascular injury, and exhibited less oxidative damage than WT mice, suggesting a role of ang II/AT1R pathway in longevity (Benigni et al., 2009). Previous studies demonstrated that β-arrestin biased ligands, that selectively block ang II binding to AT1R, simultaneously activate β-arrestin signaling pathways (Whalen et al., 2011; Wisler et al., 2014). Moreover, our laboratory reported that promotion of β-arrestin-2 signaling *via* the action of biased ligands at the AT1R was able to reverse these maladaptive changes in a genetic model of heart failure with dilated cardiomyopathy (DCM; Ryba et al., 2017). We also found that biased agonism of AT1R in chronic ang II infusion in rats preserved myofilament Ca^2+^ responsiveness and prevented ang II-related maladaptation (Monasky et al., 2013). Thus, there is ample evidence that indicates that maladaptive ang II signaling mediated through AT1R also promotes beneficial adaptive signaling *via* β-arrestin (Kim et al., 2012). However, a role for β-arrestin-2 signaling in aged-related HF remains unknown.

We hypothesize that β-arrestin-2 signaling is important to maintain normal cardiac function during aging, due to its contribution to posttranslational modifications of myofilament proteins. To test this hypothesis, we used 3–6months (young) and 12–16months (old) wild-type and β-arrestin-2 knock-out (KO) mice, assessed cardiac function by echocardiography, evaluated posttranslational modification of cardiac proteins that may explain differences in function, and determined the myofilament Ca^2+^ response of detergent-extracted fiber bundles. Here, we present novel evidence showing that the lack of β-arrestin-2 during aging promotes the phosphorylation of cardiac myosin binding protein-C (cMyBP-C) at Ser302 and decreases the phosphorylation of the myosin regulatory protein (RLC). Both, increased phosphorylation of cMyBP-C at Ser302 and decreased phosphorylation of RLC in the KO mice, are likely to play an important role as part of the mechanisms for systolic and diastolic dysfunction during aging.

## Material and Methods

### Mouse Lines

All protocols were approved by the Animal Care and Use Committee of the University of Illinois at Chicago. Transgenic β-arrestin-2 knock-out mice (KO) were previously generated in a C57BL/6 background (Walker et al., 2003) and rederived into an FVB/N genetic background. KO and FVB/N (WT) controls, young (3–6months) and old (12–16months), both males and females were used for acquisition of echocardiographic data, pCa-tension relation studies, and Western blotting analysis. protein kinase C epsilon (PKC_Ɛ_) transgenic mice were previously generated on an FVB/N background (Goldspink et al., 2004). The transgenic mice express constitutively active (A159E) PKC_Ɛ_ driven by a mouse α-myosin heavy chain promoter (Goldspink et al., 2004). Male and female PKC_Ɛ_ mice were aged to 12–15months and used to perform Western blot analysis.

### Myofibrillar Preparation

Mouse ventricles (10–15mg) were homogenized in standard relaxing buffer (SRB: 75mm KCl, 10mm Imidazole pH 7.2, 2mm MgCl_2_, 2mm EGTA, and 1mm NaN_3_) at a 1: 10 ratio relative to tissue weight. Samples were homogenized at 4°C using the Bead Ruptor 24 Elite Homogenizer (Omni International, 19-040E, Kennesaw, GA) at the following settings: power: 5m/s, time: 15s, 3cycles, and a 3min dwell time between each cycle. Homogenized samples were split into two equal samples, with one sample for preparation of myofibrils and the other sample kept as whole homogenates. To de-membranate and purify the myofibrils 1% (v/v), Triton-X 100 was added to the SRB (SRB-X 100) and added to the sample at 1:10 relative to original tissue weight (Solaro et al., 1971) then vortexed and centrifuged at 15,000 × g at 4°C for 1min. The myofibril pellets were resuspended and incubated in 500μl of SRB X-100 at 4°C and then vortexed every 5min for 15min. Myofibrils were spun down at 15,000 × g at 4°C for 1min, and the pellet was re-suspended in SRB Triton X-100 and incubated and centrifuged as before for 15min and 1min, respectively. The myofibrils were washed with 500μl of SRB and centrifuged at 15,000 × G at 4°C for 1min. The pellet was resuspended with Industrial Sample Buffer (ISB: 8M urea, 2M thiourea, 50mm Tris pH 6.8, 3% SDS, 75mm DTT, and 0.05% bromophenol blue; Fritz et al., 1989) at a 1:5 ratio relative to tissue weight. Concentrations were determined using the Pierce 660nm Protein Assay with the addition of the IDCR reagent (Thermo Fisher Scientific, 22,660, Rockford, IL). Samples were stored at −80°C.

### Gel Electrophoresis

Samples were loaded into a 12% or 15% SDS-PAGE, 0.5% bis-acrylamide, and pH 8.8 (Fritz et al., 1989). The gel ran at 200V for 75min in Tris-Glycine running buffer (0.025M Tris Base, 0.192M Glycine, and 0.1% SDS) in a criterion cell (Bio-Rad Inc., 1,656,001, Hercules, CA).

### Assessment of Myofilament Protein Phosphorylation by Pro-Q Diamond Stain

To detect overall phosphorylation changes, a 15% SDS-PAGE gel was stained with Pro-Q Diamond Phosphoprotein Gel Stain (Invitrogen, P33300, Carlsbad, CA) and destained with Pro-Q Diamond Phosphoprotein Gel Distaining Solution (Invitrogen, P33310, Carlsbad, CA). The gel was then stained with Coomassie G-250 Stain (Bio-Rad Inc., 1,610,786, Hercules, CA) to determine total protein levels. Gel images were captured with ChemiDoc MP (Bio-Rad, Inc., 13,036, Hercules, CA) and band densities were determined and analyzed by using ImageLab 6.0.1 software and Microsoft Excel.

### Western Blotting Analysis

One dimensional SDS-PAGE gels were transferred into polyvinylidene difluoride membrane for 90min at 20V in a criterion tank blotter (Bio-Rad, Inc., 13,036, Hercules, CA). Blots were blocked in either 5% nonfat dry milk + TBS-T (Tris-buffered saline, pH 7.5, and 0.1% Tween-20) or 2% bovine serum albumin (BSA) + TBS-T for 1h at room temperature. All primary antibodies were incubated overnight at 4°C. The following primary antibodies were diluted in 2% BSA + TBS-T: p-PKA-C (T197) at 1:1000 (Cell Signaling Technology, 5661S Danvers, MA) and PKA-C at 1:1000 (Cell Signaling Technology, 5842S Danvers, MA). The following primary antibodies were diluted 5% nonfat dry milk + TBS-T: p-Cdc42/Rac1 (Ser 71) at 1:250 (Cell Signaling Technology, 2,461, Danvers, MA), Cdc42 at 1:1000 (Abcam, Inc., ab64533, Cambridge, MA), RLCv at 1:2000 (Enzo Life Sciences, ALX-BC-1150-5-L001, Farmingdale, NY), actin at 1:2000 (Sigma-Aldrich A4700 St. Louis, MO), and cMyBP-C phospho-specific rabbit polyclonal antibodies (S273P, S282P, and S302P) that were generously provided by Sakthivel Sadayappan, PhD (University of Cincinnati College of Medicine, Cincinnati, Ohio). Total mouse monoclonal cMyBP-C antibody was from Santa Cruz Biotechnology (Dallas, Texas; #SC-137181) diluted to 1:2,500. Rabbit secondary (Cell Signaling Technology, 7074S, Danvers, MA) was diluted to 1:20,000 and the mouse secondary (Cell Signaling Technology, 7076S, Danvers, MA) was diluted to 1:25,000 in either 2% BSA + TBS-T or 5% nonfat dry milk + TBS-T. Secondary antibodies were incubated for 90min at room temperature. The blots were developed with Clarity ECL Substrate (Bio-Rad, Inc., 1,705,061, Hercules, CA) or Super-Signal West Femto Maximum Sensitivity ECL Substrate (Thermo Fisher Scientific, 34,094, Waltham, MA). Band densities were determined and analyzed using ImageLab 6.0.1 software (Bio-Rad, Inc., Hercules, CA) and Microsoft Excel. In cases where stripping of antibodies from the membrane was necessary, as in the case when both phospho and total (pan) antibodies were from the same species, we stripped the membranes in (6M guanidine hydrochloride, 0.3% (v/v) NP-40, and 5mm TCEP) a modified buffer (Yeung and Stanley, 2009) for 10min at room temperature and then washed in milli-Q water and repeated two additional times. After the final water wash, the blots were washed in TBS-T and blocked as described above. To verify stripping prior to re-probing, the membrane was incubated with HRP conjugated secondary antibody only and exposed to ECL to determine if the primary antibody was removed.

### Echocardiography

We used the Vevo 2,100 system (FUJIFILM VisualSonics, Toronto, Ontario, Canada) with an MS550 probe 30μm resolution to perform echocardiography, as previously described (Batra et al., 2021). Mice were previously anesthetized with 2.5–3% isoflurane in 100% O_2_ and placed in the warming plate to maintain body temperature close to 37°C during the procedure. Isoflurane concentration was adjusted to maintain a heart rate in the range of 380–460 beats per minute. We obtained two dimensional, M-mode, color-flow Doppler, and tissue Doppler images. All measurements and calculation were averaged from three consecutive cycles. Data analysis was performed using Vevo Analytic Software (VisualSonics, Toronto, ON, Canada).

### Skinned Fibers

Tension as a function of pCa (−log [Ca^2+^]) was measured in detergent-extracted (skinned) fiber bundles as previously described (Ryba et al., 2019). Mice were anesthetized with Ketamine/Xylazine (200mg/20mg/Kg body weight) and hearts were extracted following guidelines of the Veterinary Medical Association Panel on Euthanasia Guidelines. Fiber bundles, approximately 250μm in diameter and 2–4mm in length, were dissected from left ventricular papillary muscles of WT and KO mice in high relaxing (HR) buffer, pH 7.0 (10mm EGTA, 41.89mmK-Prop, 100mm BES, 6.75mm MgCl_2_, 6.22mm Na_2_ATP, 10mm Na_2_CrP, 5mm NaN_3_, and ionic strength 150mm). The following protease inhibitors were added to HR and pCa 4.5 solutions: pepstatin A, leupeptin, and phenylmethylsulfonyl fluoride. Cellular membranes of the fiber bundles were extracted using 1% Triton X-100 in HR for 30min at room temperature. Following detergent extraction, fibers were incubated in HR solution and force measurements were performed over a range of pCa values. The range of pCa values was 8.0–4.5, which was generated by mixing varying ratios of HR solution with a solution containing 10mm CaCl_2_ (pCa 4.5). The skinned fiber bundles were mounted with cellulose-acetate glue between a force transducer and a micro-manipulator. The sarcomere length was adjusted in HR to 2.3μm using HeNe laser diffraction. Initially, fibers were immersed in HR and then incubated in pCa 4.5 to generate maximum force and then placed back into the HR solution. After consecutive immersion in HR, fibers width and diameter were measured at three points. Fibers were then subjected to sequential solutions (pCa 8–pCa 4.5) to activate force development. Isometric force was measured and recorded on a chart recorder. Tension (mn/mm^2^) was determined from measurements of the fiber cross-sectional area. All experiments were performed at 23°C.

### Statistical Analysis

Statistical analysis was performed using SPSS version 25 (IBM SPSS Statistics, IBM Corporation, Armonk, New York) and GraphPad Prism 8.0.2 Software (GraphPad, Inc., San Diego, CA) was used to create the graphs. We used two-way ANOVA with a *post-hoc* LSD. The values *p* of less than 0.05 were considered statistically significant. Only the biologically relevant significances were shown in the figures. Data were presented as means ± SEM.

## Results

### Disruption of β-arrestin-2 Signaling Resulted in Impaired Systolic and Diastolic Function During Aging

**Figure 1:**
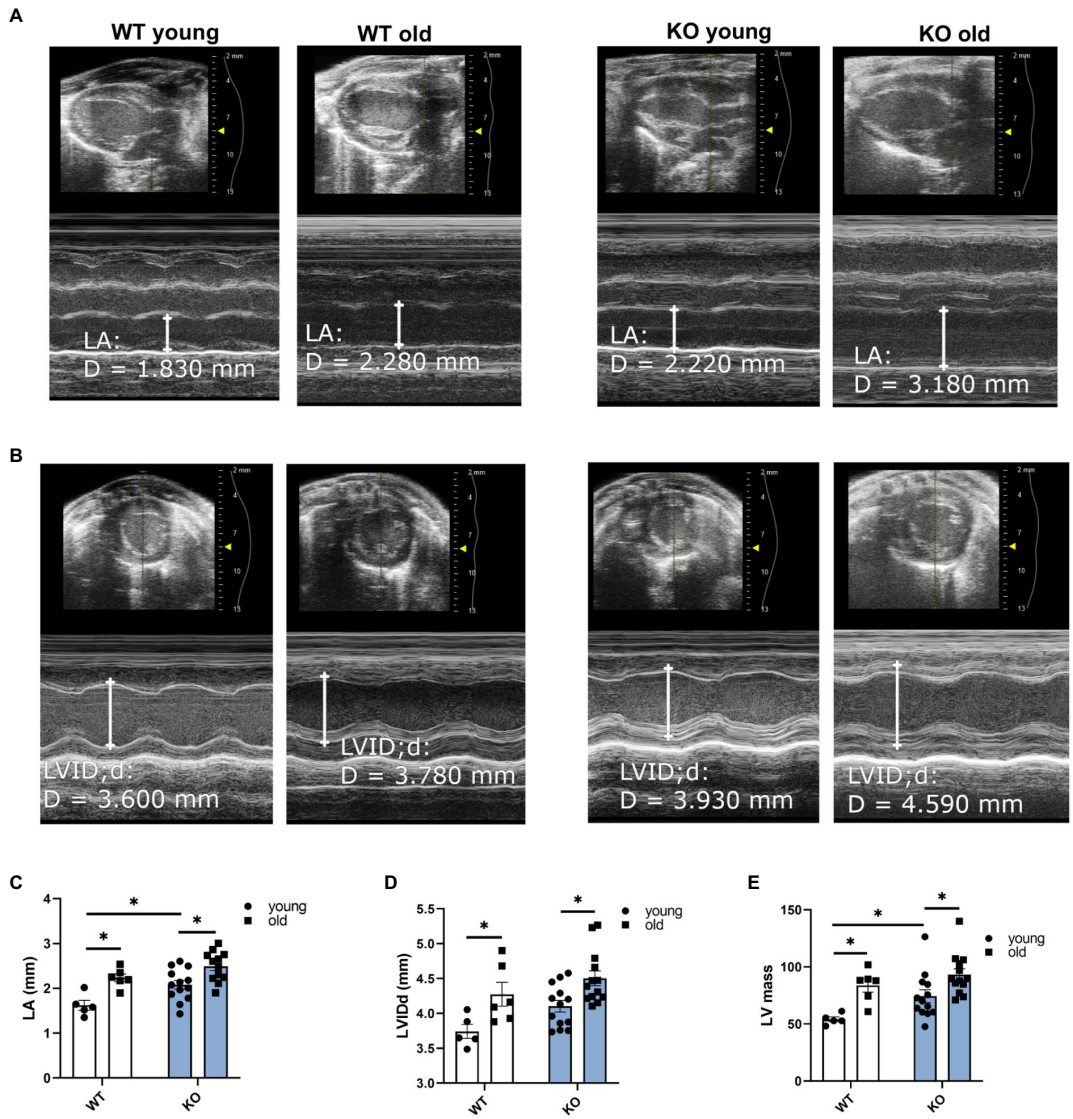
Deletion of β-arrestin-2 causes enlargement of left atria and left ventricle in aged mice. Representative B-mode and M-mode long-axis images of (A) left atrium (LA) diameter and short-axis images of **(B)** left ventricular internal diameter during diastole (LVIDd) in young and aged WT and KO mice. Quantification of **(C)** LA and **(D)** LVIDd shows enlargement in both aged WT and KO mice. **(E)** Both aged WT and KO show increased left ventricular (LV) mass. Young: 3–6months and old: 12–16months. *N*=5–13. Data presented as mean ± SEM. Outliers were identified with the ROUT method and excluded from analyses. Data analyzed with a two-way ANOVA *post-hoc* Fisher’s LSD test. ^*^p < 0.05.

**Figure 2:**
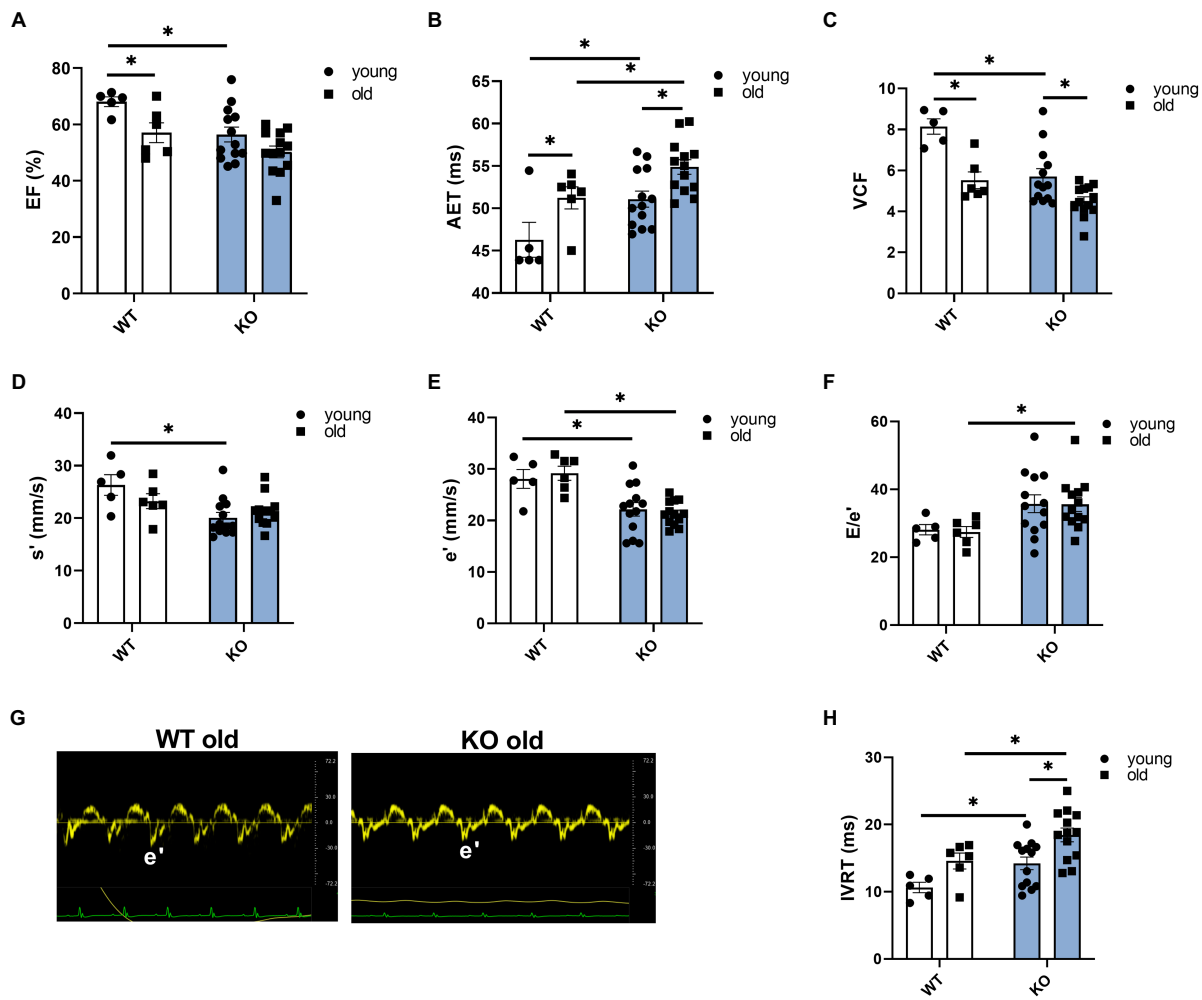
Deletion of β-arrestin-2 signaling causes impaired systolic and diastolic function in aged mice. **(A)** All mice show ejection fraction (EF)≥50%. **(B)** Aortic ejection time is prolonged in aged KO. **(C)** Circumferential fiber shortening velocity calculated as minor axis shortening/ejection time is slower in aged KO mice. **(D)** KO mice show decreased peak myocardial contraction velocity, s′. **(E)** Young and old KO mice show slower peak myocardial relaxation velocity, e′. **(F)** Aged KO mice show increased peak blood inflow velocity E/e′ ratio. **(G)** Sample tissue Doppler traces show slower myocardial relaxation velocity (e′) in aged KO mice. **(H)** Aged KO mice show prolonged isovolumetric relaxation time. Young: 3–6months and old: 12–16months. *N*=5–13. Data presented as mean ± SEM. Outliers were identified with the ROUT method and excluded from analyses. Data analyzed with a two-way ANOVA *post-hoc* Fisher’s LSD test. ^*^p < 0.05.

We used echocardiography to study the effects of β-arrestin-2 deletion on cardiac function in young (3–6months) and aged (12–16months) WT and KO mice. Figure 1A shows representative long-axis B-mode and M-mode images of left atrium (LA) and Figure 1B shows short-axis B-mode and M-mode representative images of left ventricular (LV) internal diameter during diastole (LVIDd). Both aged WT and KO mice showed enlarged LA (Figure 1C) and enlarged LVIDd (Figure 1D). Similarly, both WT and KO mice exhibited increased left ventricular mass (LV mass) during aging (Figure 1E). All mice groups maintained an ejection fraction (EF) of more than 50% (Figure 2A), but young WT mice exhibited a higher EF compared to other groups. Other systolic parameters, such as aortic ejection time (AET; Figure 2B), were prolonged in aged KO mice; whereas velocity of circumferential fiber shortening (VCF; Figure 2C), calculated as the fractional shortening of the LV [(LVIDd – LVIDs)/(LVIDd)] over ejection time, was decreased in aged KO mice. Peak myocardial contraction velocity (s′) (Figure 2D) was decreased in both young and old KO mice. Relaxation parameters, such as peak myocardial relaxation velocity during early diastolic filling (e′) (Figures 2E,G), were slower in both young and old KO mice. Old KO mice exhibited higher blood flow Doppler (E) to e′ ratio (E/e′) (Figure 2F). Additionally, aged KO mice exhibited prolonged isovolumetric relaxation time (IVRT; Figure 2H).

### Disruption of β-arrestin-2 Signaling Altered the Phosphorylation Status of Important Regulatory Myofilament Proteins

**Figure 3:**
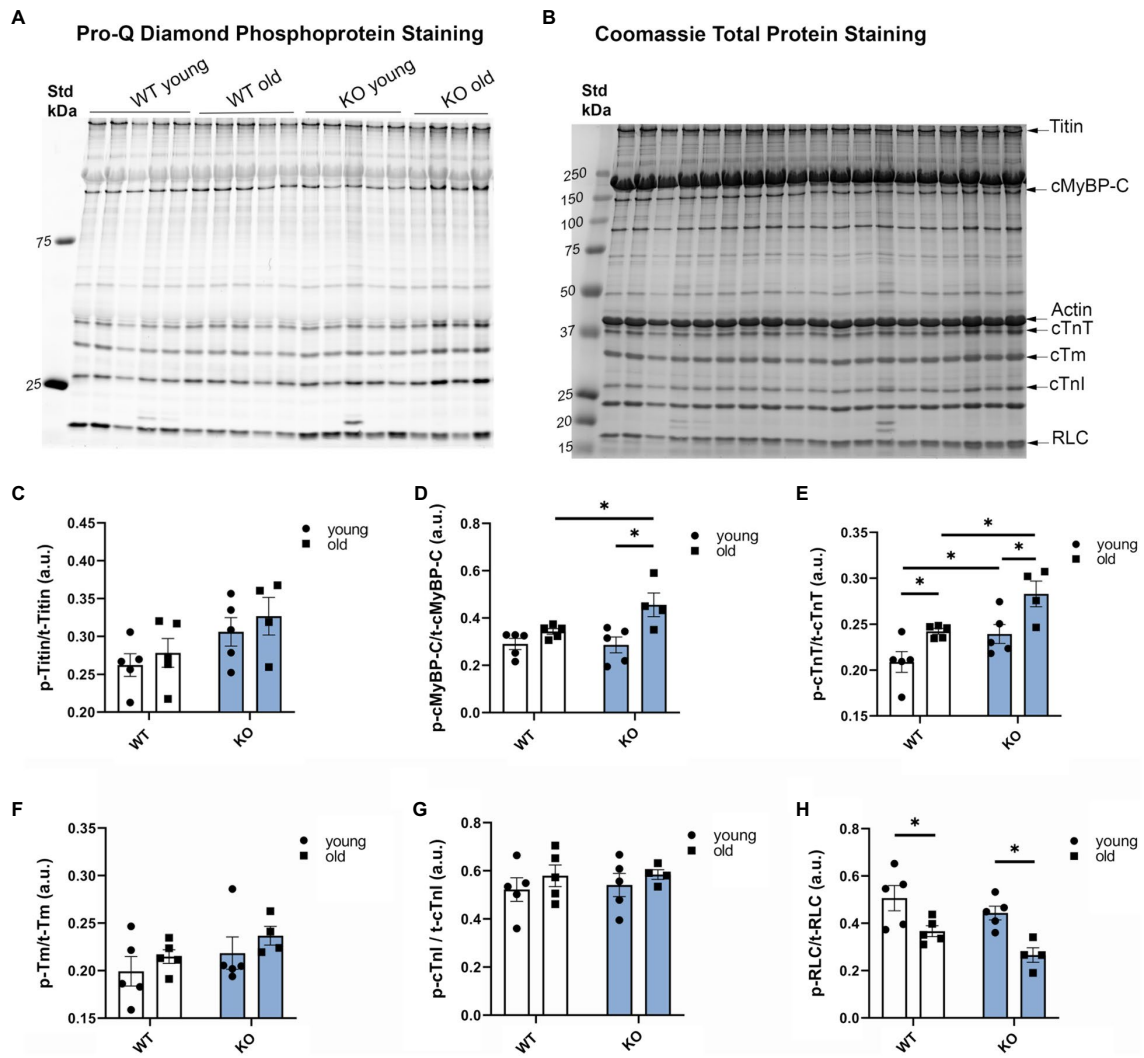
Deletion of β-arrestin-2 alters the phosphorylation of myofilament proteins. **(A)** Pro-Q Diamond staining of phosphorylated myofilament proteins on 15% SDS-PAGE gel. **(B)** Coomassie staining of total protein of the corresponding bands on the same gel. **(C)** Quantification of phosphorylated titin, **(D)** cardiac myosin binding protein-C (cMyBP-C), **(E)** cardiac troponin T, **(F)** cardiac tropomyosin (cTm), **(G)** cardiac troponin I (cTnI), and **(H)** regulatory light chain (RLC) in arbitrary units (a.u.). Young: 3–6months and old: 12–16months. *N*=4–5. Data presented as mean ± SEM. Outliers were identified with the ROUT method and excluded from analyses. Data analyzed with a two-way ANOVA *post-hoc* Fisher’s LSD test. ^*^p < 0.05.

To determine the effects of β-arrestin-2 deletion on the phosphorylation of myofilament proteins, we used Pro-Q diamond phospho-specific staining (Figures 3A–H). We found that aged KO mice showed the highest increase in phosphorylation levels of cMyBP-C (Figures 3A,B,D) and cardiac troponin T (cTnT; Figures 3A,B,E). Moreover, aged KO mice exhibited lower levels of myosin RLC phosphorylation (Figures 3A,B,H). No changes were observed in other myofilament proteins, such as titin, tropomyosin (Tm), or troponin I (TnI). These results indicate that deletion of β-arrestin-2 signaling affects downstream phosphorylation of myofilament proteins, resulting in an alteration of cardiac relaxation-contraction dynamics. To determine whether the changes in myofilament protein phosphorylation had an impact on myofilament Ca^2+^ sensitivity, we measured pCa-steady-state tension relations in detergent-extracted fiber bundles from young and old WT and KO hearts. However, we did not find significant differences in either myofilament Ca^2+^ sensitivity, as measured by the Ca^2+^ concentration at half-maximal tension (pCa_50_) or in maximal tension between groups (Supplementary Figure 1).

### Phosphorylation Levels of cMyBP-C at Ser302 Are Increased in Aged KO Mice

**Figure 4:**
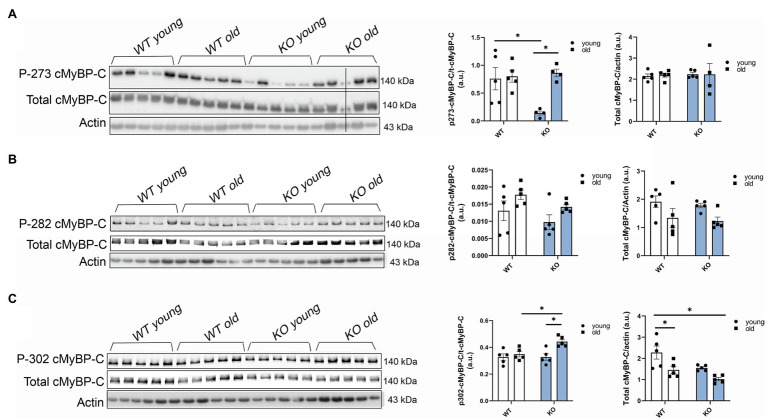
Phosphorylation of cMyBP-C at Ser302 increases in aged KO mice. Western blot images showing phosphorylation of cMyBP-C at **(A)** Ser273, **(B)** Ser282, and **(C)** Ser302 with summarized quantification and actin as loading control. Third sample on the KO old group in (A) was excluded from analyses due to loading problems as indicated with vertical black line drew across bands. t-cMyBP-C: total cMyBP-C. Young: 3–6months and old: 12–16months. Data presented as mean ± SEM. *N*=4–5. Outliers were identified with the ROUT method and excluded from analyses. Data analyzed with a two-way ANOVA *post-hoc* Fisher’s LSD test. ^*^p < 0.05.

**Figure 5:**
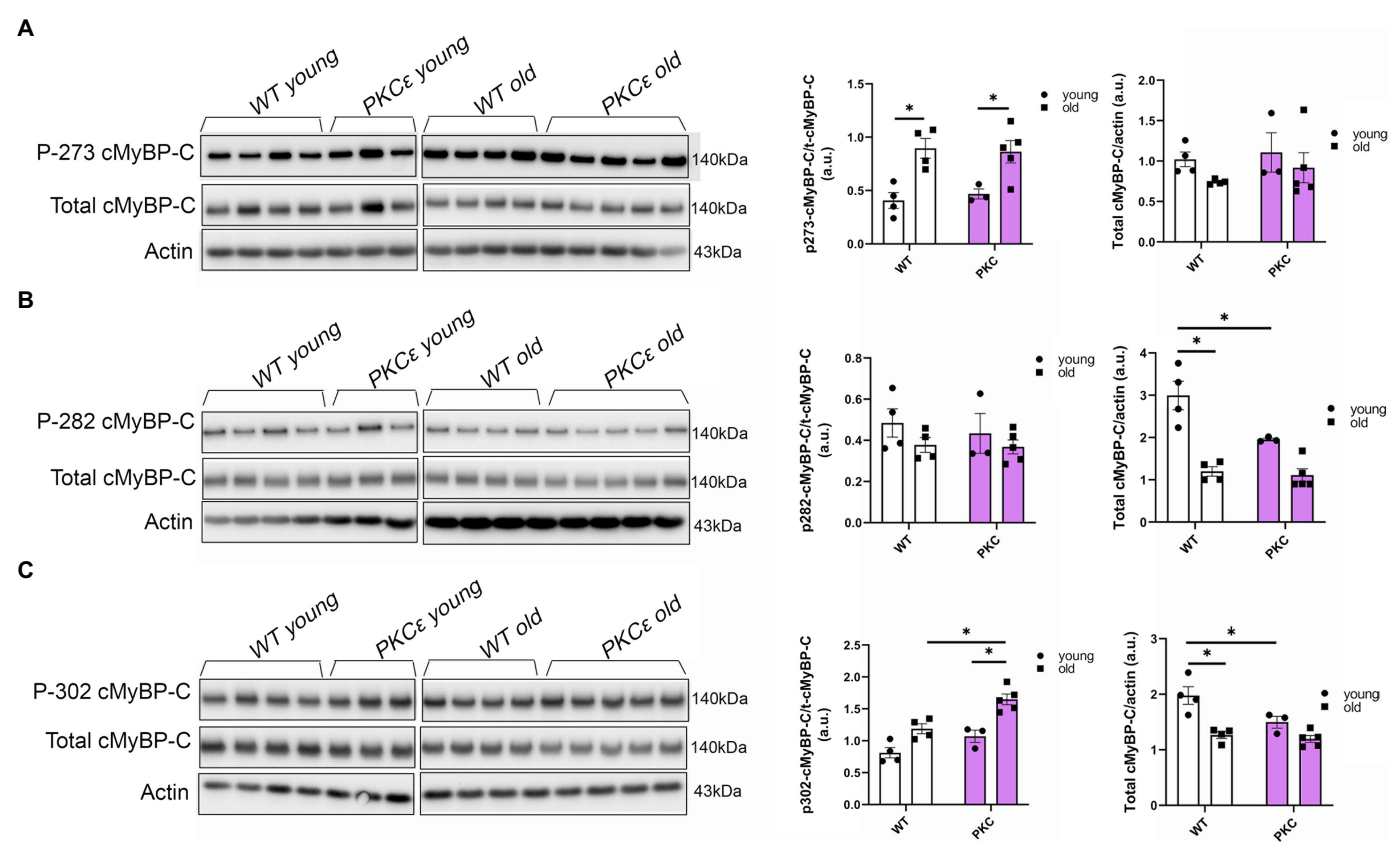
Phosphorylation of cMyBP-C at Ser302 is increased in a PKC_ɛ_ overexpression mouse model. Western blot images show phosphorylation of cMyBP-C at **(A)** Ser273, **(B)** Ser282, and **(C)** Ser302 with summarized quantification and actin as loading control. t-cMyBP-C: total cMyBP-C. Young: 3–6months and old: 12–16months. Data presented as mean ± SEM. *N*=3–5. Outliers were identified with the ROUT method and excluded from analyses. Data analyzed with a two-way ANOVA *post-hoc* Fisher’s LSD test. ^*^p < 0.05.

To further determine which cMyBP-C phosphorylation sites contributed to the overall increased phosphorylation status of cMyBP-C, we performed Western blot analyses (Figures 4A–C) and determined that aging significantly increased cMyBP-C phosphorylation at Ser302 in KO mice (Figure 4C). Interestingly, we also found that cMyBP-C phosphorylation at Ser273 was significantly reduced in the young KO mice when compared to the young WT group and the old KO group; however, aged KO mice exhibited phosphorylation levels similar to those in the other groups (Figure 4A). No other relevant differences were found at cMyBP-C Ser282 (Figure 4B). Thus, we consider the increased levels of cMyBP-C phosphorylation at Ser302 in aged KO hearts as an important factor that contributes to cardiac dysfunction in this group of mice. Importantly, among other kinases, cMyBP-C phosphorylation at Ser302 is a PKC_Ɛ_ target which its activation is negatively regulated by β-arrestin-2 signaling (Lymperopoulos et al., 2019). Thus, deletion of β-arrestin-2 signaling in our KO mice may result in increased activation of PKC_Ɛ_ in the heart. In order to determine the direct effects of PKC_Ɛ_ over-activation on cMyBP-C phosphorylation, we used a mouse model that overexpresses constitutively active PKC_Ɛ_ in the heart and performed Western blot analyses to detect cMyBP-C phosphorylation (Figures 5A–C). We found that cMyBP-C phosphorylation at Ser273 increased in both WT and PKC_Ɛ_ overexpression mice, during aging (Figure 5A). Most importantly we found that, similarly to our aged KO mice, PKC_Ɛ_ overexpression model exhibited significantly higher levels of cMyBP-C phosphorylation at Ser302, when aged to 12–15months (Figure 5C). Again, no differences were found in the phosphorylation levels of Ser282 (Figure 5B).

### Phosphorylation Levels of PKA and Cdc42 Are Increased in Aged KO Mice

**Figure 6:**
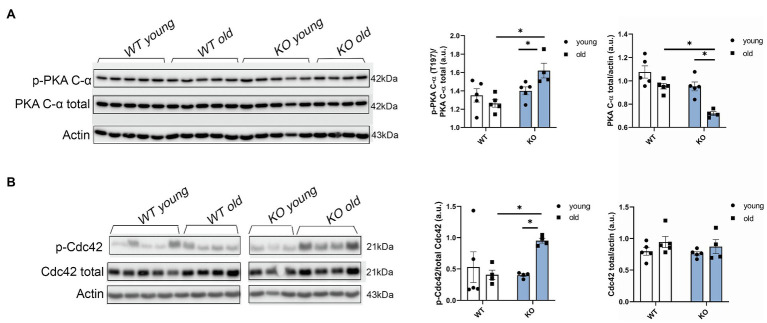
Protein kinase A (PKA) phosphorylation and Cdc42 phosphorylation are increased in aged KO mice. **(A)** Phosphorylation of catalytic (C)-α subunit at Thr197, total PKA, and actin as loading control was detected by Western blot. Aged KO mice show increased quantification of PKA phosphorylation at Thr197 over total PKA. **(B)** Representative images of the phosphorylation levels of Cdc42 at Ser71 detected by Western blot. Due to a strong phospho Cdc42 signal that was difficult to strip and both phospho and total antibodies being from rabbit, we used a separate blot to detect total Cdc42 and actin as loading control. Quantification of Cdc42 phosphorylation at Ser71 over total Cdc42 shows increased phosphorylation levels in aged KO mice. Young: 3–6months and old: 12–16months. Data presented as mean ± SEM. *N*=4–5. Outliers were identified with the ROUT method and excluded from analyses. Data were analyzed with a two-way ANOVA *post-hoc* Fisher’s LSD test. ^*^p < 0.05.

In view of our results showing that other myofilament proteins, such as RLC, were affected by the deletion of β-arrestin-2 signaling, we studied upstream signaling pathways related to β1 adrenergic signaling that were also reported to interact with β-arrestin-2 signaling (McCrink et al., 2017). We found that phosphorylation of the catalytic (C) subunit of protein kinase A (PKA) at Thr197 was significantly higher in aged KO mice (Figure 6A). Higher phosphorylation levels of PKA in its activation loop (residues 191–197) are correlated with higher PKA catalytic activity (i.e., serine/threonine protein kinase activity), as previously demonstrated (Taylor and Kornev, 2011). Moreover, we found that phosphorylation of Cdc42 at Ser71, downstream from PKA, was also increased in the aged KO group (Figure 6B). As previously demonstrated, increased Cdc42 phosphorylation levels result in decreased Cdc42 activity (Forget et al., 2002; Schwarz et al., 2012) that may affect the phosphorylation of downstream myofilament proteins, such as RLC.

## Discussion

We propose a novel concept that β-arrestin-2 signaling is essential for normal aging. Previous studies reported that with advanced aging, there is an increased expression and functional impact of β-arrestin signaling and GPCR scaffold proteins, such as G-protein linked receptor kinases (Schutzer et al., 2001; Bychkov et al., 2008). Using analyses of interactome metadata, investigators concluded that β-arrestin-2 signaling has a stronger connection to aging compared to β-arrestin-1(van Gastel et al., 2018). These are interesting findings regarding physiological adaptations during aging and raise the argument as to whether these are positive adaptations to compensate for aging stress or if these are the cause of aging-related maladies. Thus, to explore the relationship between β-arrestin-2 signaling and cardiac health during aging, we used β-arrestin-2 KO mice and aged them to 12–16months. By echocardiography, we found that aged KO mice exhibited increased LA and LV diameters (Figures 1A–D), together with increased LV mass (Figure 1E). Importantly, aged KO mice also exhibited depressed contractility parameters, such as prolonged AET (Figure 2B) and decreased VCF (Figure 2C). Moreover, aged KO mice also showed impaired relaxation parameters with slower e′ (Figures 2E,G), higher E/e′ (Figure 2F), and prolonged IVRT (Figure 2H). These functional changes correlated with decreased RLC phosphorylation, increased cTnT phosphorylation, and increased cMyBP-C phosphorylation at Ser302 (Figures 3, 4). It was previously suggested that RLC phosphorylation is affected by aging (Markandran et al., 2021). Despite, there are no statistical differences in RLC phosphorylation between aged WT vs. aged KO mice (Figures 3A,B,H); there is a trend of decreased phosphorylation in the aged KO mice group (*p*=0.07) suggesting that the deletion of β-arrestin-2 in aged mice, besides aging, contributes even more to decreased levels in RLC phosphorylation.

Although there were changes in phosphorylation between myofilaments in aged WT hearts and aged KO, we could not detect changes in the pCa-tension relations between these two groups (Supplementary Figure 1). We think a possible explanation is that the depression in RLC phosphorylation, which has been demonstrated to decrease myofilament Ca^2+^ sensitivity (Scruggs and Solaro, 2011), may be offset by an effect of cMyBP-C phosphorylation at Ser302, which has been demonstrated to slow down cross-bridge cycling resulting in a prolonged duty cycle and increased Ca^2+^ sensitivity. This effect of modification of cMyBP-C has been reported in studies deleting the N-terminal domain in which there is enhanced cross-bridge cycling and reduced myofilament Ca^2+^ sensitivity (Napierski et al., 2020). Moreover, reductions in myosin kinetics associated with reduced PKA-dependent phosphorylation and with S-glutathionylation of MyBP-C have been reported to increase myofilament Ca^2+^ sensitivity (Lovelock et al., 2012; Chakouri et al., 2018). Thus, we speculate that the net effect of the RLC and MyBP-C phosphorylation would be no change in the pCa50 for myofilament activation. This interpretation emphasizes the effects of cMyBP-C phosphorylation on myosin kinetics in control of relaxation kinetics in aging as previously reported (Rosas et al., 2015).

**Figure 7:**
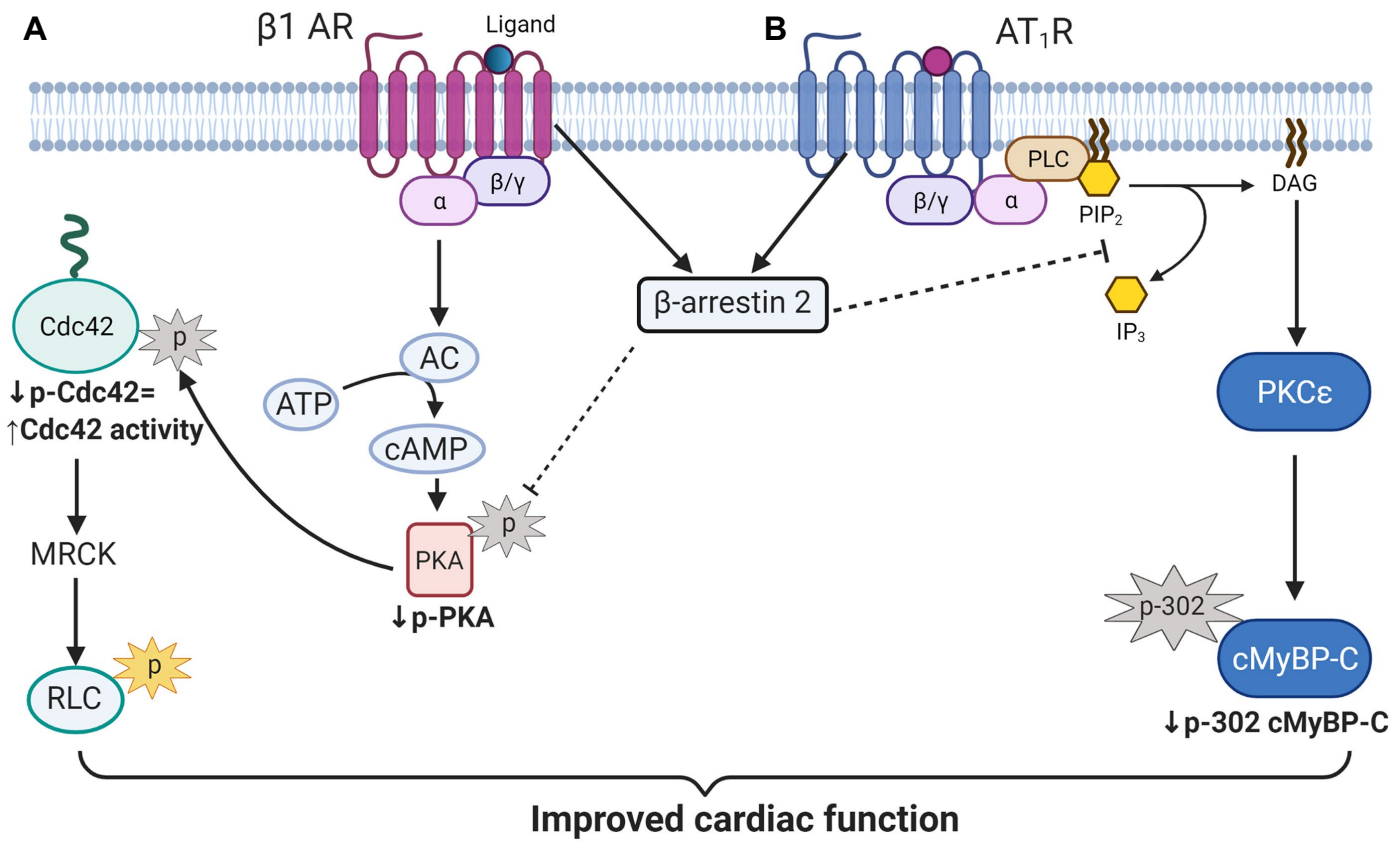
Scheme illustration of proposed β-arrestin-2 signaling in cardiomyocytes. **(A)** β-arrestin-2 signaling inhibits PKA activation by phosphorylation. Decreased PKA activity results in decreased Cdc42 phosphorylation that promotes its activation. Active Cdc42 may promote RLC phosphorylation through myotonic dystrophy-related Cdc42-binding kinase (MRCK) activation to preserve contractile function in aged mice. (**B)** β-arrestin-2 also inhibits phospholipase C (activated by AT1R signaling) resulting in the inhibition of PKC_ɛ_ signaling. Inhibition of PKC_ɛ_ activity results in decreased cMyBP-C phosphorylation at Ser302 that may prevent the heart from failing. AC, adenylate cyclase; MRCK, myotonic dystrophy-related Cdc42-binding kinase; RLC, regulatory light chain; PLC, phospholipase C; PIP2, Phosphatidylinositol 4, 5-bisphosphate; IP3, inositol triphosphate; DAG, diacylglycerol; and cMyBP-C, cardiac myosin binding protein-C. Created with BioRender.com.

One of the novel findings reported here is the demonstration of an increase in phosphorylation of Cdc42 in hearts of aged KO compared to controls. Moreover, our studies uncovered a mechanism for this increased Cdc42 phosphorylation *via* increased phosphorylation of PKA catalytic (C) subunit at Thr197 in aged KO mice. In the present study, we found that deletion of β-arrestin-2 resulted in increased activation by phosphorylation of PKA (Thr197; Figure 6A) that is associated with increased phosphorylation of Cdc42 at Ser71 (Figure 6B). It has been reported that the PKA activation loop (residues 191–197) which includes Thr197 must be phosphorylated for the C subunit to be catalytically active (Taylor and Kornev, 2011). Moreover, PKA phosphorylation of Rho small GTPases, such as Cdc42 and RhoA, significantly increases their interaction with GDI (guanine nucleotide dissociation inhibitor) forming inactive complexes that translocate from the plasma membrane to the cytosol (Forget et al., 2002). Thus, increased Cdc42 phosphorylation in aged KO mice would likely result in a suppression of Cdc42 activity. These results fit with a study employing Ingenuity Pathways Analysis, in which Van Gastel et al. identified common pathways between β-arrestin-2 and signaling by Rho family GTPases and cAMP-mediated signaling, among others (van Gastel et al., 2018). Others have shown that β1-AR-stimulated β-arrestin-2 signaling resulted in increased cardiac contractility (McCrink et al., 2017); directly by stimulating SERCA2a activity (Kho et al., 2011); or indirectly by leaving the β1-AR-stimulated cAMP-dependent signaling intact (McCrink et al., 2017). We propose that increased Cdc42 activity, resulting from an intact β-arrestin-2 signaling in WT mice, may promote RLC phosphorylation (Figure 7A). An intermediate candidate for the activation of RLC by Cdc42 may be the myotonic dystrophy-related Cdc42-binding kinase (MRCK; Unbekandt and Olson, 2014; Zhao and Manser, 2015; Figure 7A); however, further studies are needed to establish this hypothesis. Similarly, we previously reported that DCM mice treated with β-arrestin-2 biased ligands showed increased RLC phosphorylation (Ryba et al., 2017).

Decreased phosphorylation of RLC is not the sole mechanism to explain decreased contractility in our aged KO mice. Pro-Q diamond results showed increased total phosphorylation of cMyBP-C in aged KO mice when compared to other groups (Figure 3). To determine which of the phospho-sites contributed to the overall increased in cMyBP-C phosphorylation, we ran Western blot analyses and found that Ser302 site exhibited higher phosphorylation in the aged KO mice when compared to other groups (Figure 4). Moreover, we found that Ser273 phosphorylation levels decreased in the young KO group; however, Ser273 phosphorylation levels in the old KO group were similar than the other WT groups. This indicates that deletion of β-arrestin-2 during aging changes the phosphorylation pattern of cMyBP-C by a mechanism in which the phosphorylation of Ser302 is upregulated. We previously reported that cMyBP-C phosphorylation at Ser273 and Ser282 decreased with aging in a transgenic cMyBP-C (tWT) mouse model generated by re-introducing wild-type cMyBP-C into a cMyBP-C^(−/−)^ KO background generated in an SVE-129 genetic background (Rosas et al., 2019). Surprisingly, in the present study, we did not observe the same patterns in the phosphorylation of cMyBP-C in WT mice during aging; moreover, we found increased phosphorylation of cMyBP-C at Ser273 in aged WT mice (Figure 5A) and we attribute these differences to the different genetic background of mice used in both studies. Likewise, others have shown increased overall phosphorylation of cMyBP-C during aging using C57BL/6 mice (Kane et al., 2020). However, these studies did not determine specific cMyBP-C phosphorylation sites. Additionally, there are various reports pointing out that genetic background affects the cardiovascular phenotype of different murine models (Berthonneche et al., 2009; van den Borne et al., 2009; Jelinek et al., 2018).

Previous studies demonstrated that phosphorylation of cMyBP-C at Ser302 mediates cardiac responses during β-adrenergic stress, enhancing contractility (Mamidi et al., 2017). However, in our KO model, increased phosphorylation at Ser302 is correlated with decreased contractility. The phosphorylation of other sites of cMyBP-C might also be upregulated and contributed to the overall increased phosphorylation observed with Pro-Q (Figure 3). For example, some studies have implicated Ser308 as an inhibitory site (Ponnam et al., 2019). These studies showed that phosphorylation of Ser308 by PKC decreased the phosphorylation rate of other sites and reduced the overall phosphate incorporation into cMyBP-C by PKA (Ponnam et al., 2019). Although we did not examine the phosphorylation status of Ser308; importantly, both sites (i.e., Ser302 and Ser308) are PKC_Ɛ_ targets. PKC_Ɛ_ is a PKC isoform activated by lipid cofactors, such as diacylglycerol (DAG) in a Ca^2+^-independent manner (Steinberg, 2012). The classic G protein/phospholipase C-β signaling pathway, which is inhibited by β-arrestin-2(Lymperopoulos et al., 2019; Figure 7B), promotes the hydrolysis of phosphoinositide (PIP2) to generate DAG and inositol (IP3). Thus, β-arrestin-2 inhibits the activation of PKC_Ɛ_ by DAG. Transgenic mice with increased activity of PKC_Ɛ_ achieved by overexpression of a constitutively active PKC_Ɛ_ isoform (Takeishi et al., 2000) or by expression of a cardiac specific PKC_Ɛ_ activator (Mochly-Rosen et al., 2000) exhibited cardiac hypertrophy. Importantly, samples from human failing hearts exhibited increased activation of multiple PKC isoforms (Bowling et al., 1999). The activation of PKC_Ɛ_ seems to be an important factor in ventricular hypertrophy *via* phosphorylation of proteins in the costameres (Russell et al., 2010). Also, an increased concentration of PKC_Ɛ_ was found in aortic banding in rats (Gu and Bishop, 1994), guinea pigs (Takeishi et al., 1999), and in severe human aortic stenosis (Simonis et al., 2007). Most recently, it has been suggested that PKC_Ɛ_ inhibition attenuates pathological remodeling in hypertension-induced heart failure by preventing cardiac mast cell degranulation (Palaniyandi et al., 2008). Thus, PKC_Ɛ_ has been generally related to cardiac hypertrophy and changes in myofilament protein phosphorylation. Previous studies in our laboratory demonstrated that overexpression of PKC_Ɛ_ in 12months mice resulted in elevated phosphorylation levels of cTnT and cTnI and diminished phosphorylation of RLC with no changes in cMyBP-C phosphorylation (Goldspink et al., 2004). We found similar changes in our aged KO mouse model (i.e., increased phosphorylation levels of cTnT and decreased RLC phosphorylation); however, we also found increased cMyBP-C phosphorylation at Ser302. To determine if overexpression and over-activation of PKC_Ɛ_ was directly correlated with increased phosphorylation of cMyBP-C at Ser302, we used a mouse model that overexpresses constitutively active PKC_Ɛ_ in the heart. Similar changes in the phosphorylation of cMyBP-C were observed in this mouse model that exhibited increased phosphorylation at Ser302 at 12–15months of age (Figure 5). We previously reported that overexpression of constitutively active PKC_Ɛ_ induces dilated cardiomyopathy in mice when aged to 12 months (Goldspink et al., 2004). Thus, it is probable that during aging, β-arrestin-2 signaling is an important element to restrain PKC_Ɛ_ activation and prevent the heart from failing. Deletion of β-arrestin-2 signaling in our KO mice predominantly affected aged mice, resulting in changes in the phosphorylation of myofilament proteins through the unrestricted activity of PKC_Ɛ_ that promoted the phosphorylation of cMyBP-C at Ser302 (Figure 4C). As a proof of concept, overexpression of constitutively active PKC_Ɛ_ results in a similar increased phosphorylation of cMyBP-C at Ser302 (Figure 5).

In summary, our study provides novel evidence that β-arrestin-2 signaling plays an important role in the preservation of cardiac function during aging. Aging stress may induce an adaptive signaling mediated through β-arrestin-2. As a proof of concept, deletion of β-arrestin-2 affects the aging heart leading to contractile and diastolic dysfunction. Thus, failure to activate the compensatory β-arrestin-2 signaling pathway during aging may result in aged-related heart failure as seen in our KO model.

## Data Availability Statement

The raw data supporting the conclusions of this article will be made available on request to the corresponding author.

## Ethics Statement

The animal study was reviewed and approved by the Office of Animal Care and Institutional Biosafety.

## Author Contributions

AC, AB, CW, and PR performed and analyzed the biochemical experiments and manuscript editing. AC performed and analyzed the skinned fibers experiments. SC and PR collected and analyzed the echocardiography data. PR and AC prepared and wrote the manuscript. BW, RS, and PR were involved in experimental design, data evaluation analysis, and manuscript editing. All authors contributed to the article and approved the submitted version.

## Conflict of Interest

The authors declare that the research was conducted in the absence of any commercial or financial relationships that could be construed as a potential conflict of interest.

RS is a member of the Scientist Advisory Board of Cytokinetics, Inc., a consultant for Pfizer, Inc., Myokardia Inc., and a consultant to Amgen as a member of their Heart Failure Advisory Board.

## Publisher’s Note

All claims expressed in this article are solely those of the authors and do not necessarily represent those of their affiliated organizations, or those of the publisher, the editors and the reviewers. Any product that may be evaluated in this article, or claim that may be made by its manufacturer, is not guaranteed or endorsed by the publisher.
