# Supplementary material for: B-arrestin-2 Signaling Is Important to Preserve Cardiac Function During Aging
Source: Front Physiol. 2021 Aug 27;12:696852. doi: 10.3389/fphys.2021.696852 (PMC8430342; doi:10.3389/fphys.2021.696852)
Supplement: Supplementary file 1 [file Image_1.pdf]

SUPPLEMENTARY INFORMATION

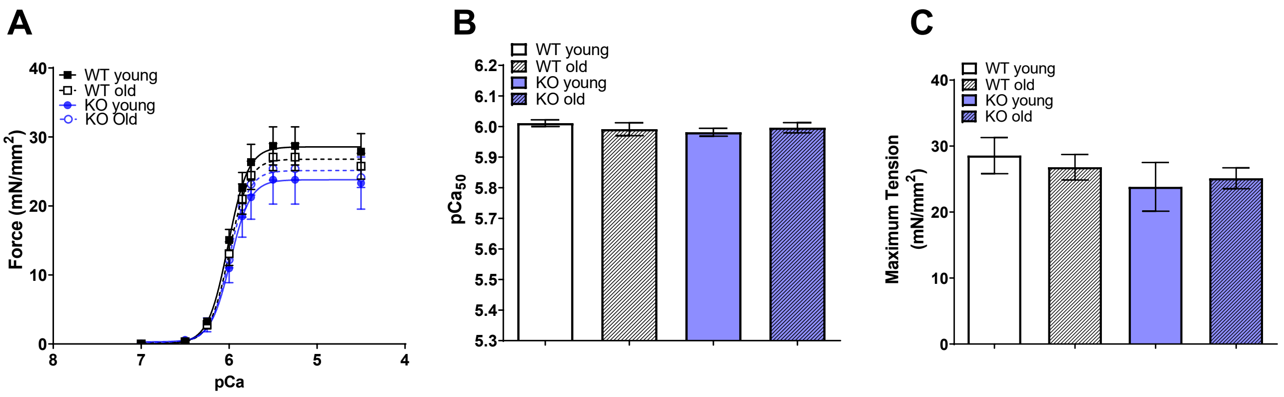

Supplementary Figure 1. Comparison of Ca^{2+} tension relations in skinned fiber bundles is similar in all groups. (A) Ca^{2+} tension relations of fibers isolated from young and old WT and KO mice. **(B)** Half-maximal activating Ca^{2+} concentrations (pCa50) are similar between groups. **(C)** Maximum tension developed in isolated fibers are similar between groups. Data were analyzed by two-way ANOVA pos hoc LSD test and represented as mean ± SEM. N=3-6 hearts per group, 5-11 fibers per group.
